# ER-α36 prevents high glucose-induced cellular senescence and apoptosis in renal tubular cell

**DOI:** 10.3389/fendo.2025.1426854

**Published:** 2025-06-09

**Authors:** Dehai Yu, Ling Luo, Yu Liang, Huili Zhou, Yinghui Xiao, Xingna An, Yingzhao Wang, Zhonggao Xu, Weixia Sun, Wanning Wang

**Affiliations:** ^1^ Department of Core Facility, The First Hospital of Jilin University, Changchun, Jilin, China; ^2^ Department of Nephrology, The First Hospital of Jilin University, Changchun, Jilin, China; ^3^ Department of Neurology, Qianwei Hospital of Jilin Province, Changchun, Jilin, China

**Keywords:** diabetic kidney disease, estrogen receptor, high glucose, apoptosis, senescence, histone methylation

## Abstract

**Background:**

The estrogen-estrogen receptor (ER) system plays a significant role in the sexual dimorphism of diabetic kidney disease (DKD), yet its specific effects on renal tubular injury under diabetic conditions remain incompletely characterized.

**Methods:**

Human renal tubular (HK-2) cells were exposed to high glucose (HG) to model diabetic conditions. Cell apoptosis was quantified by flow cytometry, and cell senescence was assessed via β-Gal staining. Western blotting was performed to analyze ER-α36 expression, PI3K/AKT pathway activity, apoptosis regulators (Bcl-2, Bax, cleaved caspase-3/7), and senescence markers (P53, P21, P27, P16). The regulatory effects of ER-α36 on EZH2 and PTEN were examined, and chromatin immunoprecipitation (ChIP) was used to assess H3K27me3 modifications at the PTEN promoter.

**Results:**

HG treatment significantly induced apoptosis and senescence in HK-2 cells, concomitant with the suppression of PI3K/AKT signaling. These effects were associated with the downregulated EZH2 expression and reduced H3K27me3 enrichment at the PTEN promoter, leading to PTEN upregulation. ER-α36 overexpression partially restored PI3K/AKT signaling, attenuated cellular injury, and reversed HG-induced epigenetic changes at the PTEN locus.

**Conclusions:**

Our findings demonstrate that ER-α36 protects renal tubular cells from HG-induced damage through EZH2-mediated epigenetic regulation of PTEN and PI3K/AKT pathway activation. These results identify ER-α36 as a potential therapeutic target for DKD.

## Introduction

Diabetic kidney disease (DKD) is one of the main causes of end-stage kidney disease (ESRD) and is recognized as a public health problem worldwide. One new nationwide survey has estimated that the prevalence of DKD among patients with type 2 diabetes in China has been over 20% (1). DKD is associated with multiple disorders including hypertension, edema, proteinuria, anemia, and cardiovascular events (2–5). Currently, the commonly used clinical drugs for treating DKD include renin-angiotensin system (RAS) inhibitors, sodium-glucose cotransporter-2 (SGLT2) inhibitors, and aldosterone receptor antagonists. However, these drugs can only delay the progression of kidney disease and cannot stop the progression of the condition. Thus, investigation of the molecular mechanisms underlying DKD is crucial for the development of novel targets and strategies for the clinical therapy of DKD.

Traditionally, DKD is considered to be a type of glomerular damage; however, increasing evidence has indicated that renal tubular damage plays a key role in the pathogenesis of DKD (6). Biomarkers of proximal tubule injury have been shown to correlate with DKD progression, independent of traditional glomerular injury biomarkers such as albuminuria (7). Glucose enters renal proximal tubular after filtered by the glomeruli, and almost 100% of glucose is absorbed by renal proximal tubular epithelial cells (RPTECs) (8). Thus, RPTECs will always be in a high glucose microenvironment, especially in the state of diabetes. Long term high glucose exposure-induced cellular senescence has been identified in different tissues, such as retina, neuron, and glomerulus (9–11). Recently, multiple researches have reported high glucose may induce cellular senescence in renal tubular cell (8, 12, 13).

The occurrence and development of DKD are closely correlated with sex. Estrogens have long been recognized to play a favorable role in the progression of chronic renal diseases (14). What is interesting, several latest studies have indicated that the females are significant protected from kidney damage in a diabetic state (15–17). Estrogen takes part in the kidney protection via its receptors. The altered or dysregulated estrogen/estrogen receptors (ERs) signaling pathways may contribute to a variety of kidney diseases, including DKD (18).

ER-α36 is a 36-kDa variant of ER-α66, which is predominantly localized at the plasma membrane and in the cytoplasm (19). ER-α36 has been identified in a variety of cancer cells as well as multiple normal tissues (20–22). Different from ER-α66, there have been few studies reporting the role of ER-α36 in diabetes and its complications, even though this molecule has been shown to mediate nongenomic estrogen signaling, including activation of the PI3K/AKT and MAPK/ERK signaling pathways (23, 24), which are highly important in glucose metabolism and insulin resistance (25).

In the present study, we investigated the effects of HG on cell apoptosis, senescence, and the related proteins using renal tubular cell line HK-2. We also examined the impact of HG on the expression of ER-α36 and explored how ER-α36 regulates the expression of EZH2 and the methylation of histones in the PTEN promoter region, thereby modulating the PI3K/AKT signaling pathway and influencing HG-induced senescence and apoptosis in renal tubular cells.

## Materials and methods

### Cell culture

Human renal cortex proximal convoluted tubule epithelial cell line HK-2 was purchased from Procell (Wuhan, China) and confirmed through STR identification. HK-2 cells were cultured in normal glucose Dulbecco’s Modified Eagle Medium (DMEM, 5.5 mmol/L glucose, NG, Procell) or high glucose DMEM (30 mmol/L glucose, HG, Procell) supplemented with 10% foetal bovine serum (FBS, Procell), 100 U/ml penicillin and 100 mg/ml of streptomycin (Life-ilab, Shanghai, China), at 37 °C in a 5% CO_2_ incubator. To exclude the effect of high osmotic pressure caused by high glucose, 24.5 mmol/L mannitol was added to the NG medium. All cells used in the experiment have passed the mycoplasma test (TransGen Biotech, Beijing, China).

### Clinical samples and ethical approval

The kidney tissue samples were obtained from Type 2 diabetic patients diagnosed with DN by renal biopsy (experimental group) and living kidney transplant donors (control group). Sample collection was approved by the Ethics Committee of the First Hospital of Jilin University (Changchun, China) (Approval number: 2021–001), and all participants provided written informed consent.

### IHC staining and evaluation

IHC was performed as previously described (26). Briefly, the paraffin-embedded tissues were cut into 4 μm slices and were dewaxed and hydrated and sealed with 3% H_2_O_2_ for 10 min. The slices were then placed in 0.1% citric acid buffer of pH 6.0 for hyperbaric heating antigenic repair for 20 min and finally incubated with anti-ER-α36 primary antibody (1:100, M000803M, Abmart, Shanghai, China) at 4°C overnight. Then the slices were restored to RT, and each slice was incubated with 25 μL of HRP-labelled secondary antibody in an immunohistochemistry kit (D601037, Sangon, China) at 37°C for 30 min. DAB staining, haematoxylin restaining and gradient alcohol dehydration were performed. After observing and taking images under an optical microscope (Olympus, Japan), two pathologists blinded to the patients’ data implemented the immunoreactive score (IRS) system to evaluate the expression level of ER-α36.

### Senescence-associated beta-galactosidase staining

β-gal activity was investigated using the senescence β-Gal Staining Kit (Beyotime Biotechnology, Shanghai, China). Briefly, HK-2 cells were fixed for 15 min with 1× fixative solution and stained with staining solution in a 37°C moisture chamber for 16 h (no CO_2_). The stained cells were imaged using microscope (Olympus, Shanghai, China).

### Assessment of cellular apoptosis by flow cytometry

Cell apoptosis was assessed using the PI-Annexin V Apoptosis Detection Kit I (BD Biosciences, NJ, USA), and the apoptotic rate was determined through fluorescence-activated cell sorting (FACS, BD, NJ, USA). The different quadrants represented distinct cell states as follows: necrotic cells resided in the upper left quadrant (Q1), late-apoptotic cells resided in the upper right quadrant (Q2), early apoptotic cells resided in the lower right quadrant (Q3), and viable cells resided in the lower left quadrant (Q4). The apoptotic rate was calculated as the sum of Q2 and Q3.

### Plasmid and siRNA

Human ER-α36 CDS (NM_001328100) was synthesized by Sangon Biotech (Shanghai, China) and subcloned into a reconstructed lentiviral expression vector pLT. Puro (kept in our lab) to form a pLT-ER-α36. Puro plasmid.

Two siRNAs were designed targeting the unique 83-nt mRNA sequence of ER-α36 (GenePharma, Shanghai, China) to specifically knock down its expression. The sequences of these to siRNA are listed in **Supplementary Table S2**.

### Cell transfection

For lentiviral transfection, 293T cells were cotransfected with recombinant pLT-ER-α36. Puro plasmid and two packaging plasmids pSPAX2 and pMD2.G (SBI, CA, USA) using Lipofectamine 2000 (Invitrogen, CA, USA) according to the manufacturer’s manual. The viral particle-containing supernatant was harvested at 24 and 48 h posttransfection. Stable cell line was established by infection of HK-2 cells with lentivirus solution and selection with 2.0 μg/ml puromycin (Sangon).

Transfection of siRNA was performed using CALNP RNAi transfection reagent (D-Nano therapeutics, Beijing, China) according to the manufacturer’s instructions. Briefly, 2.5×10^5^ HK2 cells were seeded in 12-well plates, and 30 pmol of siRNA pre-mixed with the transfection reagent was added, followed by incubation at 37°C with 5% CO_2_ for 24 h.

### Western blot

HK-2 cells were lysed with RIPA buffer (Beyotime Biotechnology, Shanghai, China). After protein quantification using an Enhanced BCA Protein Assay Kit (Beyotime), same amount proteins were separated by SDS PAGE. After being incubated with the indicated primary and secondary antibodies, the immune complex signals were detected by ECL kit (Life-ilab). Antibodies used for western blot were listed as follows: ER-α36 (1:1000), (1:2000, Proteintech Group, Hubei, China), P53 (1:1000, 2524S, Cell Signaling Technology, MA, USA), P21 (1:1000, 37543S, Cell Signaling Technology), P16 (1:1000, 18769S, Cell Signaling Technology), P27 (1:1000, 3698S, Cell Signaling Technology), Caspase 3 (1:1000, 19677-1-AP, Proteintech, Hubei, China), Caspase 7 (1:1000, 27155-1-AP, Proteintech), Bcl-2 (1:1000, 3498S, Cell Signaling Technology), Bax (1:1000, 2772S, Cell Signaling Technology), PTEN (9188S, Cell Signaling Technology), PI3K (1:1000, 4249S, Cell Signaling Technology), phospho-PI3K (1:1000, CY6427, Abways, Shanghai, China), AKT (1:1000, 60203-2-Ig, Proteintech), phospho-AKT (1:1000, 66444-1-Ig, Proteintech), EZH2 (1:1000, 5246S, Cell Signaling Technology), H3K27me3 (1:1000, 9733S, Cell Signaling Technology), H3 (1:2000, 4499S Cell Signaling Technology) and β-actin (1:5000, 66009-1-Ig, Proteintech).

### Chromatin immunoprecipitation

ChIP assays were performed with a ChIP assay kit (Beyotime) based on the manufacturer’s manual. Briefly, HK-2 cells and the transfectants were fixed with 1% formaldehyde solution for 10 min at 37 °C and quenched with 0.125 M glycine for 5 min. DNA fragments ranging from 200 bp to 1000 bp were generated using sonication and pulled down by anti-H3K27me3 antibody (1:50, 9733S, Cell Signaling Technology). Normal rabbit IgG (1:50, A7016, Beyotime Technology, Shanghai, China) was used as the negative control. The crosslinked molecules were reversed and DNA fragments were purified. Finally, the precipitated DNA was analyzed by quantitative real-time PCR Assay (qRT-PCR).

### qRT-PCR assay

Quantitation of DNA enrichment was done by qPCR using Eastep qPCR Master Mix (Promega, Beijing, China) on an LineGene 9600 Plus PCR detection system (Bioer Technology, Zhejiang, China). The primers using in ChIP assay are listed in **Supplementary Table S1**.

### Inhibitors

To investigate the impact of overexpressed ER-α36 on apoptosis and senescence of HK-2 cells through the PI3K/AKT signaling pathway, PI3K inhibitor LY294002 (20 μM) was added 1 h prior to HG treatment (27, 28). Additionally, to examine how HG regulates the histone H3K27me3 of PTEN promoter via EZH2 and affects the PI3K/AKT pathway, EZH2 inhibitor GSK126 (SC0060, Beyotime, 500 nM) (29) was also added to the culture system 1 h before HG treatment.

### Statistical analysis

All experiments were independently replicated a minimum of three times. Statistical analyses were conducted using SPSS Version 19 (IBM Corp., NY, USA). Student’s unpaired t-test was employed to assess significant differences between two groups, while one-way ANOVA was utilized for comparisons involving multiple groups. A significance threshold of P < 0.05 was applied to determine statistical significance.

## Results

### HG induces cellular apoptosis and senescence in HK-2 cells

**Figure 1:**
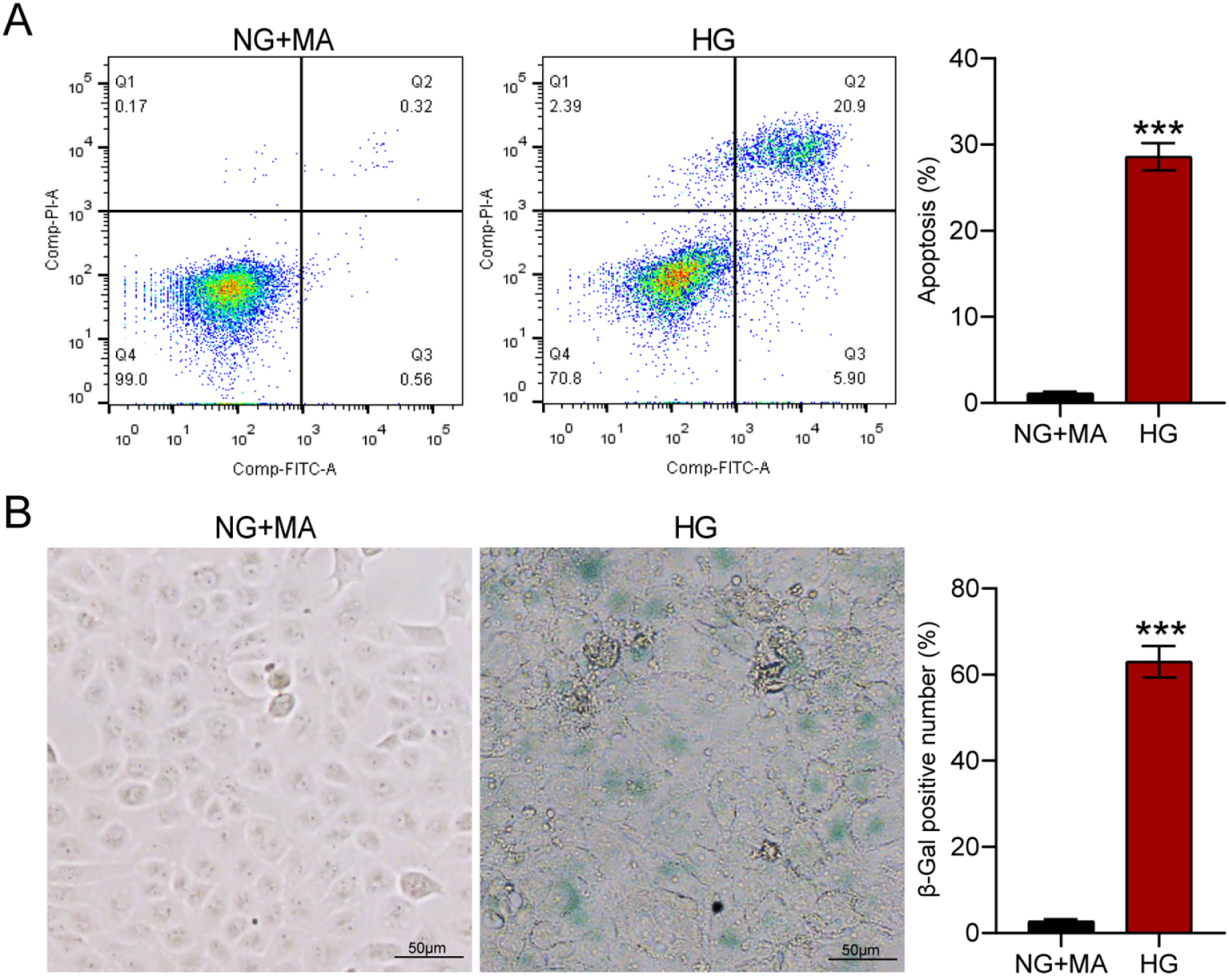
HG induces cellular apoptosis and senescence in HK-2 cells. After treatment with NG+MA or HG for 72 h, flow cytometry analysis revealed an increase in cellular apoptosis **(A)**, while SA-β-gal staining showed an increase in cellular senescence **(B)**. ***p<0.001.

To investigate the effects of HG on HK-2 cells, we examined apoptosis and senescence of HK-2 cells after HG treatment. As shown in **Figure 1**, after 72 h of HG treatment, flow cytometry revealed a significant increase in apoptosis (0.88% vs 26.8%, P<0.001, **Figure 1A**). SA-β-gal staining also showed a significant increase in the proportion of positively stained cells after HG treatment (3% vs 63%, P<0.001, **Figure 1B**). These results indicate that prolonged exposure to HG microenvironment induces apoptosis and senescence of renal tubular cells.

### HG inhibits the expression of ER-α36 and induces a memory effect

**Figure 2:**
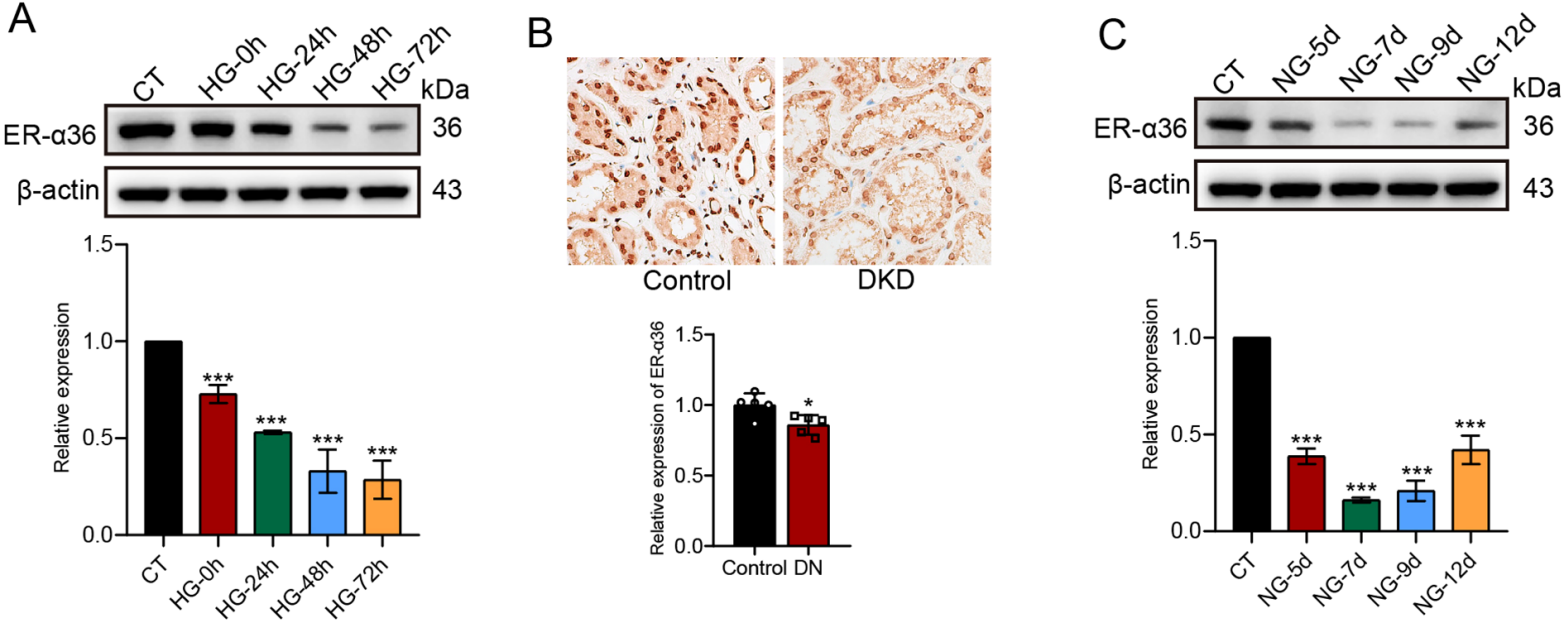
HG inhibits the expression of ER-α36 and induces a memory effect in HK-2 cells. After treatment with HG for 0, 24, 48, and 72 h, western blot analysis revealed a significant downregulation of ER-α36 expression **(A)**. IHC results also demonstrated significantly higher ER-α36 expression levels in DKD renal tissues compared to normal kidney tissues **(B)**. Following 72 h of HG treatment, the culturing medium was switched to NG medium, and ER-α36 expression was assessed by western blot on days 5, 7, 9, and 12, showing a memory effect of HG inhibition on ER-α36 **(C)**. *p<0.05, ***p<0.001.

As described above, although studies have reported the role of ER-α36 in diabetes and its complications, the exact impact of HG on the expression of this molecule and its effects on renal tubular cell behavior remain unclear. In this study, we used western blotting to assess the expression of ER-α36 after HG treatment for 0, 24, 48, and 72 h. The results revealed a significant inhibition of ER-α36 expression upon HG treatment (**Figure 2A**). As another transcript variant of ER-α, the expression of ER-α66 (66-kDa) remained unaffected by HG treatment (**Supplementary Figure S1**). We also examined ER-α36 expression in kidney tissues from DKD patients. IHC results demonstrated significantly lower ER-α36 expression levels in DKD renal tubular cells compared to normal kidney tissues (**Figure 2B**).

Following HG treatment for 72 h, we replaced the medium with NG medium and continued monitoring ER-α36 expression on days 5, 7, 9, and 12. It was observed that ER-α36 expression remained suppressed from day 5 to day 9. However, on day 12 after switching to NG medium, this inhibitory effect gradually alleviated (**Figure 2C**). This indicates the presence of HG memory effect in HK-2 cells.

### HG activates apoptotic and senescence-related signaling pathways

**Figure 3:**
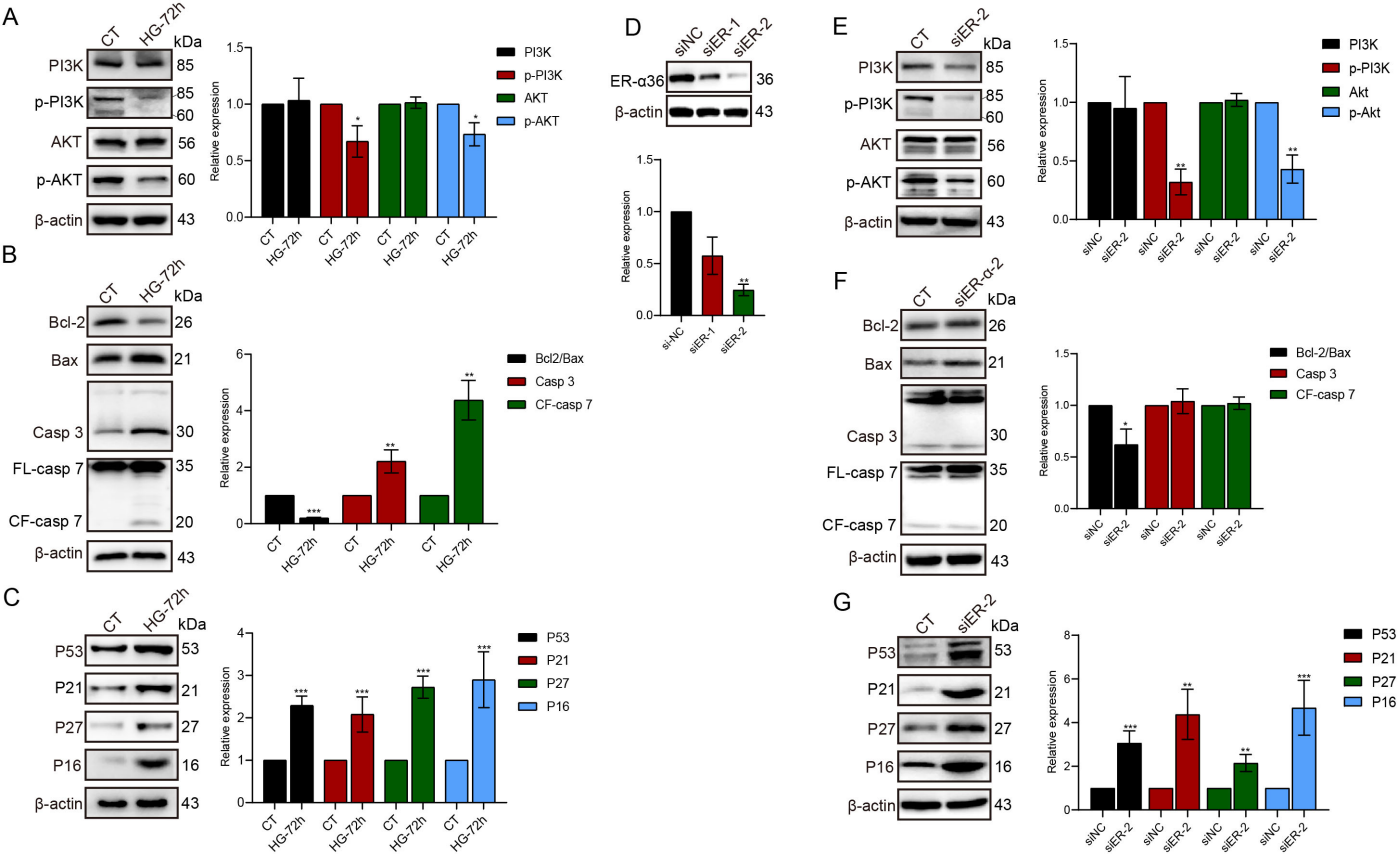
HG activates apoptotic and senescence-related signaling pathways in HK-2 cells. After 72 hs of HG treatment, the expression of p-PI3K and p-AKT was suppressed **(A)**. Additionally, the apoptotic-related protein Bcl-2 was downregulated, while the levels of Bax, Casp3, and cleaved-Casp7 were increased **(B)**. Furthermore, the expression of senescence-related proteins P53, P21, P27, and P16 was upregulated **(C)**. Knockdown of ER-α36 by siRNA **(D)** suppressed the phosphorylation of PI3K and Akt **(E)**, decreased Bcl2/Bax ratio **(F)** and upregulated P53, P21, P16, and P27 expression **(G)**. *p<0.05, **p<0.01, ***p<0.001.

To elucidate the molecular mechanisms by which HG induces apoptosis and senescence in HK-2 cells, we first examined the activation status of the PI3K/AKT signaling pathway, which is closely associated with the cellular regulatory functions of ER-α36. As shown in **Figure 3A**, after 72 h of HG treatment, there is a significant decrease in the phosphorylation levels of PI3K and AKT in HK-2 cells. We then assessed the apoptotic markers in apoptotic cells and found a significant decrease in Bcl2/Bax ratio, along with activation of Casp3 and Casp7 (**Figure 3B**). Furthermore, we examined the expression levels of senescence-associated proteins, including P53, P21, P27, and P16, and found that all of these proteins were activated by HG (**Figure 3C**).

To further demonstrate that HG-induced changes in the PI3K/Akt signaling pathway are associated with the suppression of ER-α36 expression, we knocked down ER-α36 using siRNA in HK2 cells (**Figure 3D**, **Supplementary Figure S2**) and examined the activation of PI3K/Akt as well as the expression of senescence- and apoptosis-related proteins. The results showed that p-PI3K and p-Akt expression was downregulated (**Figure 3E**), while P53, P21, P16, and P27 expression was upregulated (**Figure 3G**). Additionally, the Bcl2/Bax ratio decreased, indicating enhanced apoptosis, but no significant change was observed in caspase levels (**Figure 3F**).

**Figure 4:**
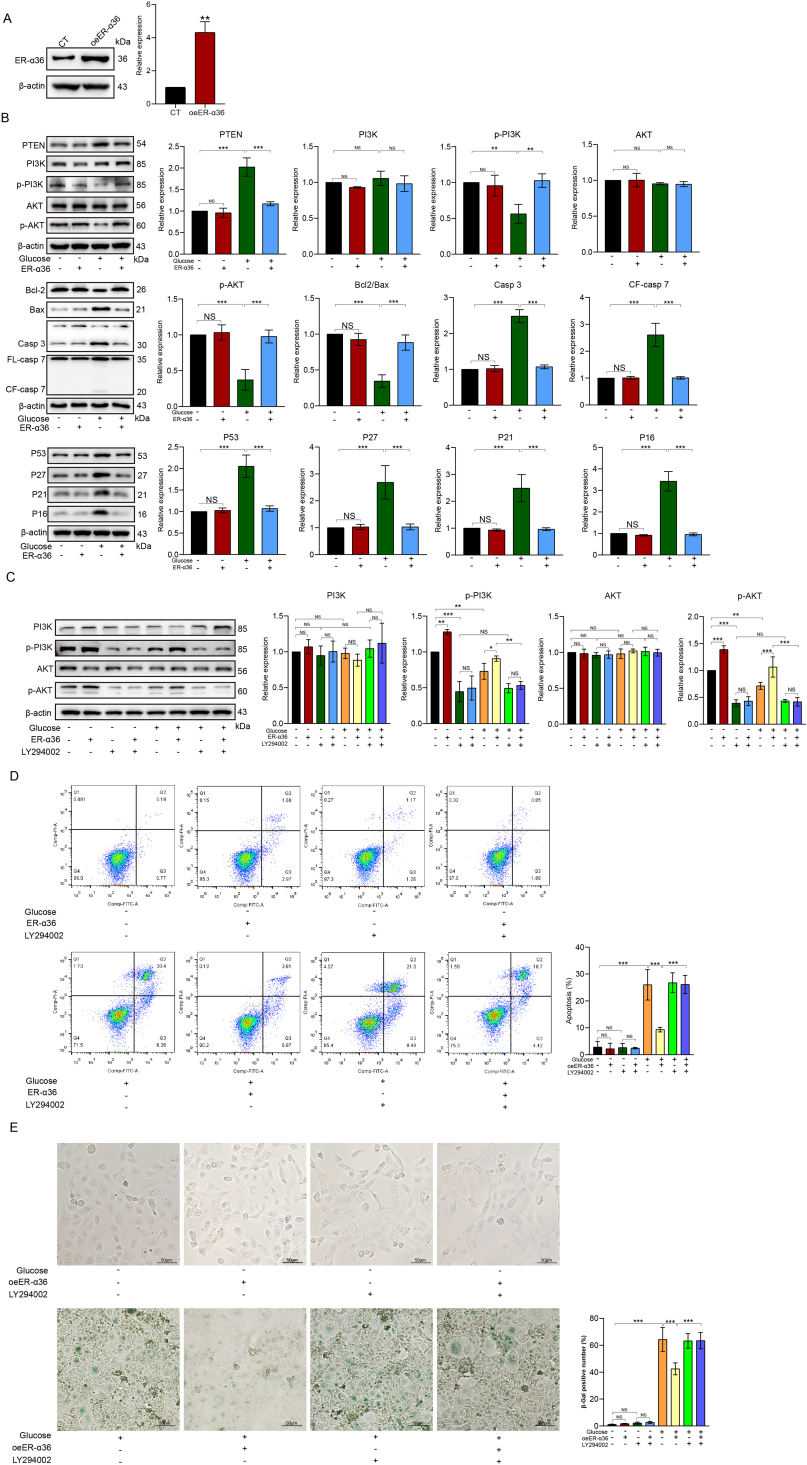
Enforcing expression of ER-α36 attenuates HG-induced apoptosis and senescence in HK-2 cells. ER-α36 was transduced into HK-2 cells via lentivirus, as confirmed by western blot analysis **(A)**. Similarly, after treating transfectants with HG for 72 h, it was observed that overexpression of ER-α36 reversed the inhibitory effect of HG on p-PI3K and p-AKT and partially suppressed the activation of apoptosis- and senescence-related proteins induced by HG. Additionally, transfection of ER-α36 also inhibited the upregulation of PTEN induced by HG **(B)**. Furthermore, when the PI3K/AKT signaling pathway was blocked using the inhibitor LY294002 **(C)**, ER-α36 failed to reverse HG-induced cellular apoptosis **(D)** and senescence **(E)**, indicating that ER-α36 influences HG-induced cellular apoptosis and senescence via the PI3K/AKT signaling pathway. *p<0.05, **p<0.01, ***p<0.001; NS, not significant.

To further clarify the involvement of ER-α36 in mediating the inhibition of the PI3K/AKT pathway by HG and activation of senescence and apoptosis signaling pathways, we overexpressed ER-α36 in HK-2 cells (**Figure 4A**) and subsequently treated the transfectants with HG, while simultaneously assessing the activation of the PI3K/AKT signaling pathway and the expression of apoptotic and senescence-related proteins. As shown in **Figure 4B**, western blotting results demonstrate that overexpression of ER-α36 in HK-2 cells significantly reverses the inhibitory effects of HG on p-PI3K and p-AKT. Furthermore, elevation of endogenous ER-α36 levels also attenuates the activation of apoptotic and senescence-related proteins induced by HG.

According to research reports, the PI3K/AKT pathway is an important signaling pathway through which HG affects cell apoptosis and senescence (30, 31). As described in the preceding results, our experiments also found that HG induces apoptosis and senescence in HK-2 cells by inhibiting the phosphorylation of PI3K/AKT. To further confirm this finding, we used a PI3K pathway inhibitor (LY294002) in HK-2 cells overexpressing ER-α36 and observed the effects on cell apoptosis and senescence after inhibitor treatment. **Figure 4C** shows the expression levels of PI3K/p-PI3K and AKT/p-AKT in HK-2 cells after HG treatment, ER-α36 transfection, and LY294002 treatment. We observed that although ER-α36 can reverse the inhibitory effect of HG on the PI3K/AKT signaling pathway, this reversal effect is interrupted by LY294002. Similarly, flow cytometry experiments and SA-β-gal staining results demonstrate that ER-α36 overexpression in HK-2 cells reverses HG-induced cell apoptosis and senescence, which is also disrupted by LY294002 (**Figures 4D, E**).

### HG inhibits the expression of EZH2 and reduces the level of histone H3K27me3 in the PTEN promoter region

**Figure 5:**
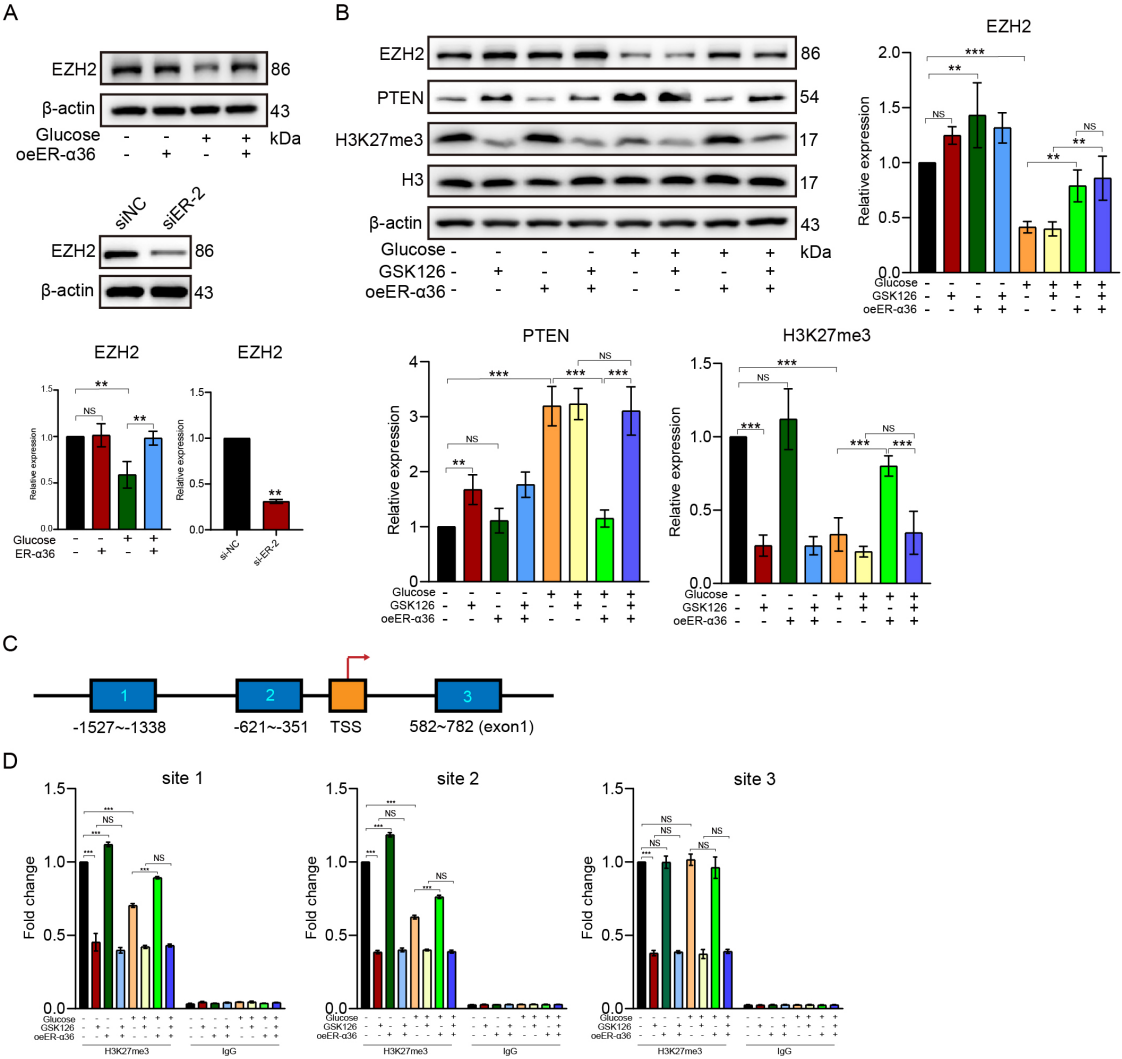
HG treatment inhibits the expression of EZH2 and decreases the level of histone H3K27me3 in the PTEN promoter region. It is observed that HG treatment can suppress EZH2 expression, while transfection of ER-α36 can reverse this process. Conversely, ER-α36 knockdown in HK2 cells further downregulated EZH2 levels **(A)**. Additionally, HG treatment is observed to inhibit H3K27me3 and upregulate PTEN expression, while transfection of ER-α36 can reverse this process. Furthermore, GSK126, the inhibitor of EZH2, can block the effect of ER-α36 on PTEN and H3K27me3 under HG conditions **(B)**. The results of ChIP experiments were consistent with the above observations **(C, D)**.

Due to the observation of the HG memory effect, we speculate that epigenetic regulation may play a role in HG-induced apoptosis and senescence of HK-2 cells. It has been demonstrated in several reports that HG induces histone methylation and regulates gene expression by affecting EZH2 level in cells (29, 32). In this study, we observed a significant downregulation trend in EZH2 expression levels in HK-2 cells after 72 h of HG induction (**Figure 5A**). However, when ER-α36 was transfected into HK-2 cells, the inhibitory effect of HG on EZH2 was significantly reversed (**Figure 5A**). Conversely, knockdown of ER-α36 in HK2 cells significantly downregulated EZH2 expression levels (**Figure 5A**).

The PTEN signaling pathway is the upstream target of the PI3K/Akt pathway (33), so we also assessed the impact of HG treatment on PTEN expression. As shown in **Figure 5B**, we found that HG treatment significantly upregulates the expression level of PTEN in HK-2 cells, while transfection of ER-α36 into cells significantly reverses the promoting effect of HG on PTEN expression. We also simultaneously examined the expression of histone H3K27me3 in HK-2 cells after HG treatment and found that HG can inhibit the expression level of H3K27me3 in HK-2 cells, while overexpression of ER-α36 can reverse the inhibitory effect of HG on H3K27me3. To confirm that HG affects the levels of H3K27me3 and PTEN by inhibiting EZH2, we treated cells with the specific EZH2 inhibitor GSK126. Western blotting results showed that GSK126 significantly inhibits the level of H3K27me3 and also abolishes the inhibitory effect of ER-α36 on PTEN in an HG environment (**Figure 5B**).

**Figure 6:**
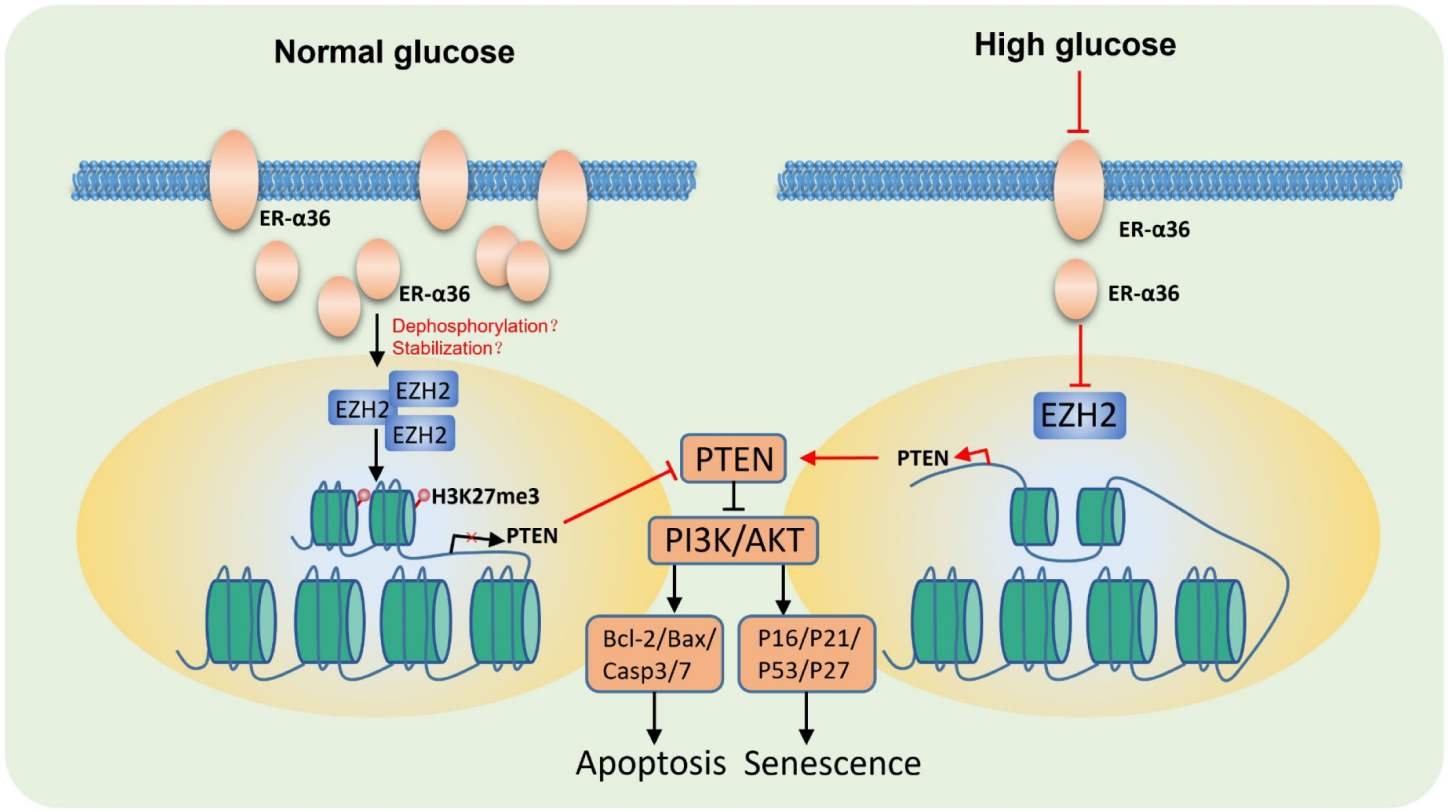
Molecular mechanism of ER-α36-mediated protection against HG-induced apoptosis and senescence. Under HG conditions, reduced expression of ER-α36 leads to downregulation of EZH2 and H3K27me3 at the PTEN promoter. Increased expression of PTEN inhibits the PI3K/AKT signaling pathway, thereby inducing cellular apoptosis and senescence.

To further explore the potential mechanism by which HG promotes PTEN expression, we designed three pairs of PCR primers targeting the PTEN promoter region and conducted chromatin immunoprecipitation (ChIP) experiments using H3K27me3 antibody (**Figure 5C**). The ChIP results showed that HG can inhibit H3K27me3 at two regions (-1527~-1338 and -621~-351) upstream of the PTEN transcription start site (TSS), but has no effect on H3K27me3 at the region (582~782) located in the first exon of PTEN. Transfection of ER-α36 significantly promotes H3K27me3 at these two regions (**Figure 5D**). These results indicate that HG promotes PTEN expression and inhibits PI3K/AKT activation by downregulating EZH2 expression levels and reducing H3K27me3 in the PTEN promoter region, ultimately inducing apoptosis and senescence of HK-2 cells (**Figure 6**).

## Discussion

Animal and clinical studies have demonstrated a link between gender and the prevalence and progression of DKD. For example, Spires et al. observed significant sexual dimorphism in various DKD-related parameters in rats, with females showing substantial protection from severe DKD development despite both genders displaying a DKD phenotype (17). Additionally, recent research by Clotet-Freixas et al. revealed that healthy male primary human PTECs exhibited increased mitochondrial respiration, oxidative stress, apoptosis, and injury when exposed to HG compared to their female counterparts (34).

The Estrogen-ER system was believed to hold significance in the sexual dimorphism of DKD. Studies have shown that estrogen deficiency could worsen renal pathological conditions (such as glomerulosclerosis and tubulointerstitial fibrosis) in diabetic rats. However, supplementation with estrogen or raloxifene (a selective estrogen receptor modulator) can mitigate these changes by decreasing lipid peroxidation and oxidative stress (35–37). Similarly, ER deficiency has also been demonstrated to exacerbate renal fibrosis in patients and animal models with chronic kidney disease (38).

The classical ER subfamily primarily comprises ERα and ERβ, both of which are members of the nuclear receptor family. ER-α36 (36 kDa) is a short isoform ERα (66 kDa) but lacks two transcriptional activation function domains (AF-1 and AF-2) (18). It predominantly localizes at the plasma membrane and in the cytoplasm (19). Up to now, the field with the most extensive reports on ER-α36 has been tumor research, such as breast cancer. It is reported that ER-α36 is highly expressed in ER-negative breast tumors, promoting cell proliferation, escaping apoptosis, and enhancing metastasis (20). Besides its involvement in regulating tumor initiation and development, multiple studies have shown that ER-α36 is also involved in regulating various cellular behaviors, such as neuroprotection, cytokine secretion, and cell development (39–41).

The induction of cell apoptosis and cellular senescence by HG has been confirmed by several studies (42, 43). Diabetic patients typically exhibit hyperglycemia, which can create a HG microenvironment for renal tubular cells. This HG environment may have various effects on renal tubular cells, including alterations in intracellular signaling pathways, inflammation and oxidative stress, changes in permeability, and fibrosis. However, as of now, there have been no reports indicating the role of ER-α36 in the process of HG-induced renal tubular pathological changes. In this study, we found that HG-induced senescence and apoptosis of renal tubular cells are associated with down-regulation of ER-α36. However, overexpression of ER-α36 can reverse the damage of HG on HK-2 cells. To our knowledge, this is the first report showing the protective effect of ER-α36 against HG-induced tubular cell injury.

ER-α36 can exert regulatory effects on cells through a non-genomic mechanism, primarily relying on the activation of several classical cell signaling pathways, including the PLC/PKCs pathway, Ras/Raf/MAPK pathway, PI3K/AKT pathway, and cAMP/PKA pathway (18). Additionally, it has been reported that HG may induce cell senescence and apoptosis through regulation of the PI3K/AKT pathway (44, 45). In this study, we demonstrate that HG induces apoptosis and senescence in HK-2 cells by inhibiting the activation of PI3K/AKT, and overexpression of ER-α36 can partially activate the PI3K/AKT pathway and attenuate HG-induced damage to HK-2 cells. Similar research has been reported by Han et al., who showed that ER-α36 exerts a neuroprotective effect against H_2_O_2_ toxicity through the PI3K/AKT and MAPK/ERK1/2 signaling pathways (39). Wang et al. also reported that HG can induce nucleus pulposus cell apoptosis and senescence, while resveratrol can attenuate HG-induced nucleus pulposus cell apoptosis and senescence through activation of the PI3K/Akt pathway (42). Our research results align with the conclusions of Wang et al.

HG can influence the methylation of histone H3K27 and regulate gene expression by affecting the expression of EZH2. However, current research results are inconsistent regarding whether HG upregulates or inhibits EZH2. Zhang et al. reported an increase in the expression levels of EZH2 and H3K27me3 in heart tissues of streptozotocin (STZ) diabetic rats or HG-treated neonatal rat cardiac fibroblasts (32). Li et al. suggested that HG may decrease the phosphorylation of EZH2 at the T311 site and increase the level of H3K27me3 in cardiac fibroblasts (29). Additionally, Oh et al., Das et al., and Sánchez-Ceinos et al. also reported that HG promotes EZH2 and H3K27me3 expression in umbilical cord blood-derived mesenchymal stem cells, mesangial cells, and aortic endothelial cells (46–48). However, Siddiqi et al. and Jia et al. found that EZH2 expression was reduced in glomeruli isolated from STZ diabetic rats and HG-treated renal mesangial cells (49, 50). In this study, we observed that HG significantly inhibits the expression of EZH2 and its downstream target H3K27me3 in renal tubular cells.

PTEN, as a crucial negative regulator of the PI3K/AKT signaling pathway, can inhibit the phosphorylation activation of AKT. Multiple studies have shown that HG can also affect the expression of PTEN—some suggest upregulation, while others demonstrate downregulation (51–53). In our experiment, we found that HG significantly upregulates the expression level of PTEN, which is consistent with the inhibition of the PI3K/AKT pathway. Additionally, EZH2 can bind to the promoter region of PTEN and increase the H3K27me3 level (54, 55). Another study conducted by our team on gliomas revealed that the histones corresponding to the sequences -1527bp~-1338bp and -621bp~-351bp on the PTEN promoter are the two main modification sites targeted by EZH2 (data unpublished). In this study, through ChIP assays, we demonstrated that HG inhibits the expression of EZH2 and reduces the levels of H3K27me3 in these two regions. This mechanism is hypothesized to underlie the HG-induced upregulation of PTEN.

In summary, in this study, we found that in renal tubular cells overexpression of ER-α36 can inhibit HG-induced cell apoptosis and senescence in renal tubular cells. Mechanistically, we discovered that HG downregulates EZH2 and the methylation of histones in PTEN promoter regions, thereby promoting PTEN expression and inhibiting the PI3K/AKT signaling pathway. Moreover, overexpression of ER-α36 can reverse this process. This study represents the first report on ER-α36’s modulation of renal tubular cell apoptosis and senescence via epigenetic regulation. Our findings further elucidate the molecular mechanisms underlying sexual dimorphism in DKD and offer a novel avenue for DKD treatment.

Notably, different researchers have provided evidence showing that HG affects cell signaling and epigenetic regulation in different ways, while also highlighting the fact that different cell types exhibit inconsistent responses to HG. Additionally, *in vitro* experiments may not perfectly simulate the response of renal tubules to hyperglycemia *in vivo*. Therefore, to draw more precise conclusions, further animal experiments are still needed.

## Data Availability

The original contributions presented in the study are included in the article/**Supplementary Material**. Further inquiries can be directed to the corresponding authors.

## Supplementary material

Supplementary Figure 1 The expression of ER-α66 was not affected by HG treatment.

Supplementary Figure 2 Specific knockdown of ER-α36 did not affect ER-α66 expression.
