# Supplementary figures and images for: ER-α36 prevents high glucose-induced cellular senescence and apoptosis in renal tubular cell
Source: Front Endocrinol (Lausanne). 2025 Jun 9;16:1426854. doi: 10.3389/fendo.2025.1426854 (PMC12183064; doi:10.3389/fendo.2025.1426854)

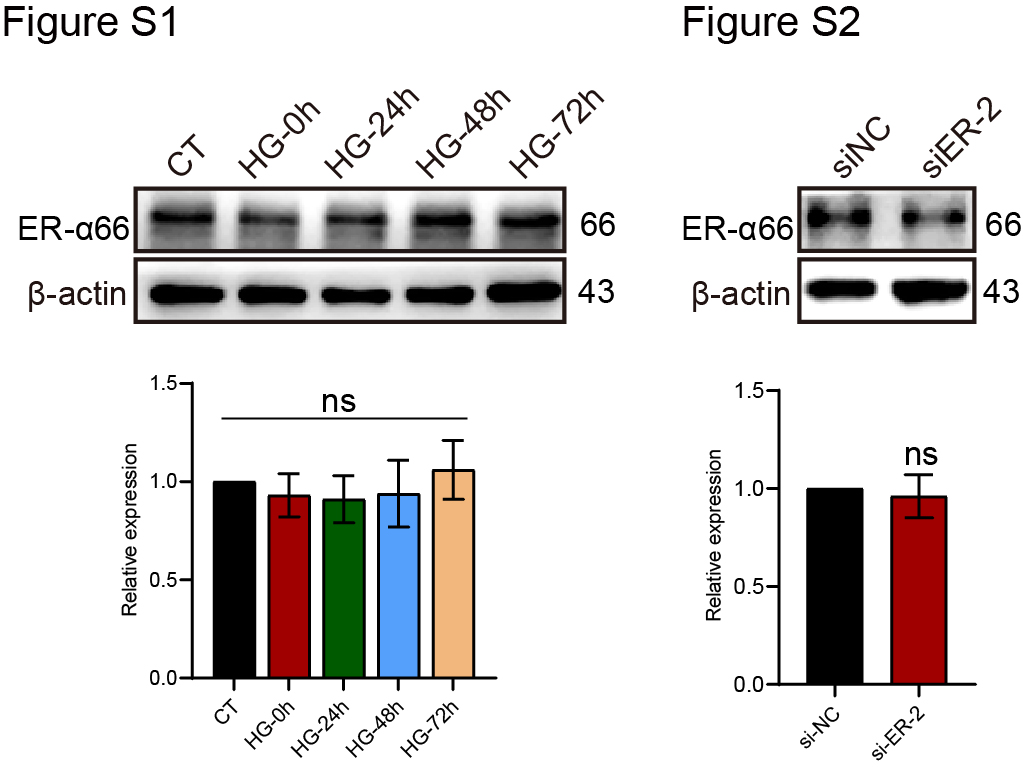

Supplement: Supplementary Figure 1 — The expression of ER-α66 was not affected by HG treatment. [file Image1.jpeg]
