# Supplementary material for: ER-α36 prevents high glucose-induced cellular senescence and apoptosis in renal tubular cell
Source: Front Endocrinol (Lausanne). 2025 Jun 9;16:1426854. doi: 10.3389/fendo.2025.1426854 (PMC12183064; doi:10.3389/fendo.2025.1426854)
Supplement: Supplementary Figure 2 — Specific knockdown of ER-α36 did not affect ER-α66 expression. [file Table1.docx]

**Supplementary Table S1.** Primer sequences for ChIP

| Gene | Sequence (5’ – 3’) |
| --- | --- |
| *PTEN* promotor Site 1 | Forward primer: CAGAATAGGTCGATGTAGAGC |
|  | Reverse primer: GGGAACTGGTTACACAAGCAC |
| *PTEN* promotor Site 2 | Forward primer: GGATGAGGTGATACACGCTG |
|  | Reverse primer: GACCACGCTGCTCAGTGTAG |
| *PTEN* promotor Site 3 | Forward primer: TCGCCTGTCACCATTTCCAG |
|  | Reverse primer: AGACGAATAATCCTCCGAACG |

**Supplementary Table S2.** Sequences of siRNAs

| siRNAs | Sequence (5’ – 3’) |
| --- | --- |
| *siRNA-*1 | GCUUAAUUCUGGUAUCUCATT |
| *siRNA-2* | CACAUGUAGAAGCAAAGAATT |
| *siNC(control)* | UUCUCCGAACGUGUCACGUTT |
